# Assessing Ozone-Related Health Impacts under a Changing Climate

**DOI:** 10.1289/ehp.7163

**Published:** 2004-08-16

**Authors:** Kim Knowlton, Joyce E. Rosenthal, Christian Hogrefe, Barry Lynn, Stuart Gaffin, Richard Goldberg, Cynthia Rosenzweig, Kevin Civerolo, Jia-Yeong Ku, Patrick L. Kinney

**Affiliations:** ^1^Mailman School of Public Health, Columbia University, New York, New York, USA; ^2^Atmospheric Sciences Research Center, State University of New York at Albany, Albany, New York, USA; ^3^Columbia University Center for Climate Systems Research, New York, New York, USA; ^4^National Aeronautics and Space Administration–Goddard Institute for Space Studies, New York, New York, USA; ^5^New York State Department of Environmental Conservation, Bureau of Air Research, Albany, New York, USA

**Keywords:** air pollution, climate change, global warming, mortality, ozone

## Abstract

Climate change may increase the frequency and intensity of ozone episodes in future summers in the United States. However, only recently have models become available that can assess the impact of climate change on O_3_ concentrations and health effects at regional and local scales that are relevant to adaptive planning. We developed and applied an integrated modeling framework to assess potential O_3_-related health impacts in future decades under a changing climate. The National Aeronautics and Space Administration–Goddard Institute for Space Studies global climate model at 4° × 5° resolution was linked to the Penn State/National Center for Atmospheric Research Mesoscale Model 5 and the Community Multiscale Air Quality atmospheric chemistry model at 36 km horizontal grid resolution to simulate hourly regional meteorology and O_3_ in five summers of the 2050s decade across the 31-county New York metropolitan region. We assessed changes in O_3_-related impacts on summer mortality resulting from climate change alone and with climate change superimposed on changes in O_3_ precursor emissions and population growth. Considering climate change alone, there was a median 4.5% increase in O_3_-related acute mortality across the 31 counties. Incorporating O_3_ precursor emission increases along with climate change yielded similar results. When population growth was factored into the projections, absolute impacts increased substantially. Counties with the highest percent increases in projected O_3_ mortality spread beyond the urban core into less densely populated suburban counties. This modeling framework provides a potentially useful new tool for assessing the health risks of climate change.

A warming climate may result in increased morbidity and mortality related to ozone, an impact that is often overshadowed by concerns about the direct effects of increased heat stress (Githeko and Woodward 2003; Kalkstein and Greene 1997; McMichael et al. 2003). Peak ambient O_3_ concentrations are typically observed in summer months, when higher temperatures and increased sunlight enhance O_3_ formation and also lead to increased emissions of biogenic and fugitive anthropogenic hydrocarbons, important precursors of O_3_ formation. Above 90°F (32°C), a strong positive association has been found between temperature and ground-level O_3_ production (Patz 2000). Numerous epidemiology studies have reported associations between O_3_ and hospital admissions or emergency visits for respiratory conditions, diminished lung function, and a variety of other health outcomes (Kinney 1999; Koken et al. 2003). A relatively recent but growing body of literature also has documented acute effects on mortality in large cities, in many cases while controlling for particulate matter and other pollutants (Dominici et al. 2003; Hoek et al. 1997; Moolgavkar et al. 1995; Thurston and Ito 2001; Vedal et al. 2003).

Summer heat waves and high O_3_ days are current health stressors in the New York metropolitan region, and their impacts may increase under a changing climate (Kinney et al., in press). Warming of 1.4–3.6°C (2.6–6.5°F) by the 2050s has been projected by the Hadley and Canadian global climate models (Columbia Earth Institute 2001). General circulation models (GCMs) such as these typically provide output (e.g., surface temperatures) at resolutions of hundreds of kilometers. Several past studies have assessed health impacts of climate change using GCMs (Anderson HR et al. 2001; Donaldson et al. 2001; Kalkstein and Greene 1997). One study in the United Kingdom used a GCM to examine potential climate impacts on O_3_-related health effects and concluded that a 10% increase in premature mortality could result by 2020, with a 20% increase possible by 2050 (Anderson HR et al. 2001).

To better assess localized impacts of climate change, models are needed that can project meteorologic parameters at scales of tens of kilometers. One strategy to accomplish this is to link GCM outputs with regional climate models (RCMs). There have been few assessments of health impacts that have used these “down-scaled” models, mostly to assess heat effects rather than air quality impacts (Dessai 2003; McMichael et al. 2003). Other knowledge gaps include potential urban versus rural differentiations in health impacts of climate change, the relative regional impacts of climate-related O_3_ versus heat impacts, and the relative contributions of various model components to overall uncertainty.

In response to the need for improved methods for assessing potential air pollution health impacts of climate change at regional scales, the New York Climate and Health Project (NYCHP) developed and tested an integrated modeling system in the New York metropolitan region (Kinney et al., in press). The modeling system employed coupled global/regional models to simulate meteorology and air quality in the 2020s, 2050s, and 2080s. The objective of the present report is to assess and compare summer O_3_-related mortality in the 1990s and 2050s. We analyzed the independent and joint effects of climate change and anthropogenic O_3_ precursor emission change on summer O_3_ concentrations and resulting mortality. We also examined the sensitivity of O_3_-related mortality to a range of modeling assumptions, including population growth and O_3_ threshold effects.

## Materials and Methods

**Figure 1:**
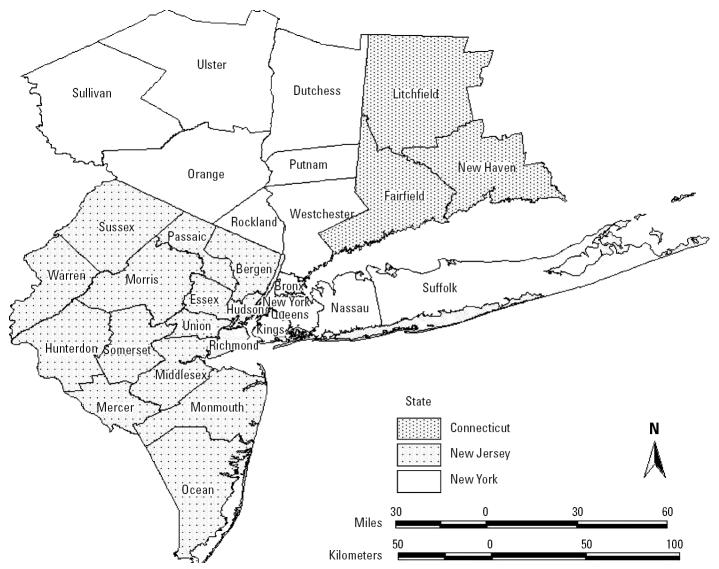
The 31-county New York metropolitan study area.

The three-state, 31-county health impact domain for this study is depicted in Figure 1. With New York City at its core, this 33,600-km^2^ (13,000 mi^2^) region is presently home to > 21 million people. It has a widely varying landscape and a range of population densities and land uses. In addition to the nation’s largest city, the metropolitan region includes a relatively pristine watershed, the source of New York City’s drinking water; substantial agricultural land in parts of northern New York, Long Island, and central New Jersey; and an estimated 1,600 cities, towns, and villages.

### Climate and O_3_ modeling.

To develop predictions of surface O_3_ concentrations on a 36-km grid over the region of interest, we linked models for global climate, regional climate, and regional air quality. Global climate modeling was carried out using the Goddard Institute for Space Studies (GISS) GCM (Russell et al. 1995), which produced simulations of hourly climate over the globe at 4° × 5° grid resolution from the 1990s through 2100. Changes in greenhouse gas emissions projections were taken from the A2 scenario of the Intergovernmental Panel on Climate Change (IPCC) *Special Report on Emissions Scenarios* (*SRES*) (Nakicenovic and Swart 2000). The A2 scenario is characterized by high carbon dioxide emissions (up to 30 gigatons/year), relatively weak environmental concerns, and large population increases (15 billion worldwide by 2100). Analyses of the more environmentally friendly *SRES* B2 scenario of growth will be the subject of future reports.

Outputs from the GISS GCM were used as inputs to an RCM that was run for the summer seasons (June–August) for five consecutive mid-decadal years (e.g., 1993–1997) in the 1990s and 2050s. Only five summers per decade could be modeled with available computer resources. The mid-decadal years were chosen arbitrarily for these model runs and were meant to be representative of each decade. Regional climate modeling was carried out using the Penn State/National Center for Atmospheric Research Mesoscale Model 5 (MM5) (Grell et al. 1994), which made it possible to simulate climate factors on a 36-km horizontal grid over the New York metropolitan area.

For O_3_ simulations, we used the Community Multiscale Air Quality (CMAQ) model (Byun and Ching 1999) with the Sparse Matrix Operator Kernel Emissions Modeling System (SMOKE) (Houynoux et al. 2000). The GCM/MM5 linked model provided the meteorologic inputs needed for the air quality simulations at a resolution of 36 km. We compared the GCM/MM5/CMAQ model outputs for the eastern United States in five summers of the 1990s with observations for the same period. The model successfully captured the observed year-to-year and shorter-term temporal variability in O_3_ as well as the spatial pattern of summer average daily maximum 1-hr O_3_ levels and the frequency distribution of extreme O_3_ events (Hogrefe et al. 2004). These O_3_ simulations did not take into account the effects of possible O_3_ precursor emission changes from outside of the modeling domain upon future air quality within the study area.

### Health impact analysis.

We used a risk assessment framework to assess changes in O_3_-related mortality in the 2050s compared with the 1990s. Although many other health outcomes have been associated with O_3_ exposures, we chose to limit the present analysis to acute effects on daily mortality for all internal causes. For each decade, county-level mortality impacts were computed as *M* = (*P*/100,000) × *B* × CRF × *E*, where *M* is the estimated number of daily deaths attributable to O_3_ concentrations; *P* is the estimated county population during time period of interest; *B* is the estimated baseline county-level daily mortality rate in June–August (per 100,000 population); CRF is the concentration–response function, which quantifies the magnitude of the proportional change in daily mortality that would be expected in response to a given daily O_3_ concentration, based on results from the epidemiologic literature; and *E* is the daily 1-hr maximum O_3_ concentrations in June–August in each county, interpolated from the GISS/MM5/CMAQ model outputs described above. To estimate the typical June–August summer mortality in each decade, the average daily O_3_-related mortality across all five summers simulated in each decade was calculated.

County populations in the mid-1990s were estimated from 2000 U.S. Census data (U.S. Census Bureau 2001). These population figures remained constant in the 2050s in base-case calculations aimed at isolating the climate influence. For sensitivity analyses in which population was allowed to grow, 2050s populations were estimated by applying the proportion of the U.S. population that each county comprised in Census 2000 to a set of A2-consistent U.S. growth projections through the year 2100 (Gaffin S, personal communication). This method projected a 53% regional population increase by 2055. This growth rate was assumed to apply equally to all counties; future work will loosen this assumption by tying population growth to results from land use modeling for the region. The population age structure was held constant at Census 2000 conditions.

Average 1990s daily summer mortality rates for each of the 31 counties in the study area were estimated as follows. Annual all-age crude mortality data for all internal causes (*International Classification of Diseases*, *9th Revision*, codes 0–799.9 for years 1990–1998, and *International Classification of Diseases, 10th Revision*, codes A00–R99 for year 1999) (Anderson RN et al. 2001) were obtained from the U.S. Centers for Disease Control and Prevention (CDC 2004) for each of the 31 counties. A scaling factor of 0.237 (the proportion of annual deaths 1993–1996 that occurred in June–August in the five boroughs of New York City) was applied to the annual mortality rates to adjust them to summer-only seasonal mortality. This was converted to a daily rate. We held baseline mortality rates constant in all analyses. Although mortality rates will undoubtedly change in future in response to changes in the age distribution of the population and in health care, projection of these shifts was beyond the scope of the present study.

CRFs from the epidemiologic literature describe the relationship between changes in daily ambient O_3_ concentrations and changes in mortality risk. A CRF for O_3_-related mortality, expressed as relative risk (RR), of 1.056 per 100 ppb increase in daily 1-hr max O_3_ [95% confidence interval (CI), 1.032–1.081] was used in our base-case analyses, taken from a pooling of seven studies that controlled well for temperature effects using nonlinear functions (Thurston and Ito 2001). Because the RR of mortality associated with an increase in O_3_ is modeled as an exponential function, the change in RR associated with a change of O_3_ (ΔO_3_) is of the form RR = exp (βΔO_3_) − 1, where β is the pooled Poisson regression slope reported by Thurston and Ito (2001), and ΔO_3_ is the incremental change in summer-seasonal mean O_3_ concentrations [adapted from Davis et al. (1997)].

### Impact assessments.

We performed two primary mortality assessments and a series of sensitivity analyses. Mortality assessment 1 (M1) estimated future O_3_ concentrations and associated changes in mortality resulting from climate change alone, where the only changes from 1990s assumptions involve altered A2 greenhouse gas emissions. Mortality assessment 2 (M2) estimated future O_3_ and mortality under A2 greenhouse gas emission assumptions along with growth in anthropogenic O_3_ precursor emissions at rates consistent with the A2 scenario. Sensitivity analyses examined alterations in several of the individual assumptions underlying the primary assessments.

#### M1: A2 climate only.

The objective here was to assess how climate change alone might contribute to changes in summer O_3_ concentrations and associated mortality in the New York region over the next 50 years, in isolation from other factors. Here, county population totals were held constant at Census 2000 levels through the 2050s (U.S. Census Bureau 2001). Similarly, anthropogenic O_3_ precursor emissions were held constant at the 1996 county-level U.S. Environmental Protection Agency (EPA) National Emissions Trends inventory; thus, no projected changes in anthropogenic precursor emissions were applied in the CMAQ projections of 2050s summer O_3_. The base case did allow for temperature-dependent changes in biogenic and mobile source emissions. For mortality estimation, we assumed no threshold for O_3_ impacts.

#### M2: A2 climate and precursors.

The objective here was to assess the potential impacts of allowing for changes in both climate and O_3_ precursor emissions. Anthropogenic O_3_ precursor emissions from the 1996 inventory were scaled up using A2 growth factors provided by the Center for International Earth Science Information Network (Nakicenovic and Swart 2000). For the 2050s, these scaling factors were oxides of nitrogen (NO_x_), an increase of 29.5%, and volatile organic compounds (VOCs), an increase of 8%. No detailed county-level or national projections of U.S. emissions taking into account the effects of emissions control programs such as NO_x_ state implementation programs and O_3_ National Ambient Air Quality Standards (NAAQS) are available at this point for the time horizon of 2050 (U.S. EPA 2004). In lieu of U.S.-specific projections of anthropogenic emissions, we used the emission projections of the IPCC *SRES* A2 marker scenario generated by the atmospheric stabilization framework socioeconomic model (Pepper et al. 1998; Sankovski et al. 2000).

The IPCC *SRES* describes various future emissions scenarios based on projections of population, technology change, economic growth, and the like, and these emission factors are superregional in nature (Nakicenovic and Swart 2000). Specifically, all countries in the Organization for Economic Cooperation and Development region OECD90 (including the United States, Canada, and Western Europe) are assumed to have the same emission growth rates. In other words, the IPCC *SRES* scenarios are not designed to reflect country-specific emission growth. For the IPCC *SRES* A2 scenario used in this study, the emissions of the O_3_ precursors NO_x_/VOCs increase by 125/60% globally and 29/8% for the OECD90 region (including the United States) by the 2050s (Nakicenovic and Swart 2000). The IPCC *SRES* A2 emission growth for the OECD90 region might be overly pessimistic given enacted or contemplated U.S. emission control programs, whereas by using these emission growth factors for the CMAQ modeling, we maintain internal consistency with the global and regional climate modeling in which the A2 greenhouse gas emissions were used. Therefore, rather than attempting to predict “realistic” air quality in the 2050s, our simulations investigate the overall effect of the A2 scenario, a possible (although pessimistic) trajectory into the future.

All other assumptions (no regional population growth; no threshold concentration for O_3_-related mortality; CRF value = RR 1.056 per 100 ppb O_3_) remained the same in mortality assessment M2 as for M1.

### Sensitivity analyses.

We performed a variety of sensitivity analyses to evaluate the effects on O_3_ mortality projections of changing individual modeling assumptions. The baseline assumptions for all of the sensitivity analyses were those described above for M1. The following sensitivity analyses were carried out: S1, population growth, climate change, and O_3_ precursor emission changes, with the objective to assess the potential impacts of a full set of A2 scenario assumptions; S2, O_3_ precursor emission changes without climate or population change; S3, climate change only but the existence of an O_3_ threshold below which no mortality effects occur is assumed; one recent O_3_–mortality study suggested the existence of a summer threshold (Kim et al. 2004); the regional minimum value (20.3 ppb) from the 31-county average of CMAQ summer 1-hr daily maximum O_3_ simulations for the 1990s was used as the threshold value.

## Results

### Primary mortality assessments.

**Figure 2:**
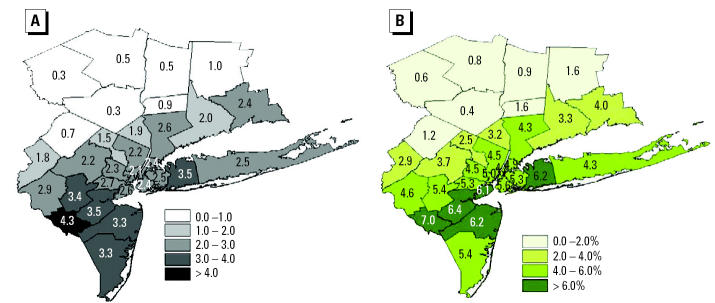
Estimated changes in O_3_ and associated summertime mortality in the 2050s compared with those in the 1990s for M1, where climate change alone drives changes in air quality. (*A*) Changes in mean 1-hr daily maximum O_3_ concentrations (ppb). (*B*) Percent changes in O_3_-related mortality.

County-specific O_3_ concentrations and associated mortality estimates under climate change alone (M1) for the 1990s and 2050s are shown in Table 1 and Figure 2. The range of projections for O_3_ mortality in each county shown in Table 1 is based on calculations that apply the lower and upper confidence limits of the Thurston and Ito (2001) 95% CI in the risk assessment. Increases in estimated summer averaged daily 1-hr maximum O_3_ concentrations ranged from 0.3 to 4.3 ppb across the 31 counties. The geographic distribution of O_3_ increases shows greater impacts in coastal counties and in those along the predominant upwind air mass trajectory from the southwest (Figure 2A). As would be expected, the distribution of percent increases in O_3_-related mortality shows the same geographic pattern (Figure 2B), although the absolute numbers of O_3_-related deaths (Table 1) are a strong function of underlying county populations. This analysis suggests that the greatest percent increases in summer O_3_ mortality will occur across the urban core and especially in a ring of suburban counties immediately surrounding the city to the southwest and east (central Long Island). Over the entire region, there was a projected median increase of 4.5% in O_3_-related deaths.

**Figure 3:**
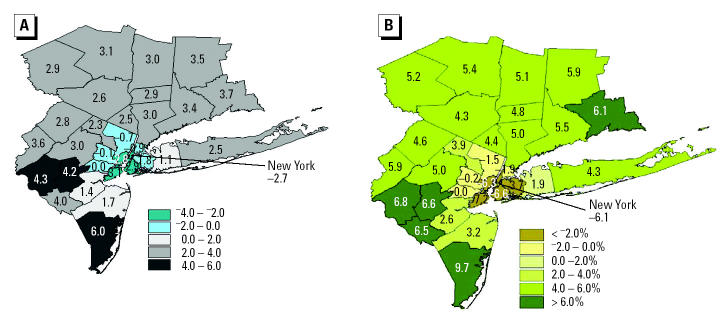
Estimated changes in O_3_ and associated summertime mortality in the 2050s compared with those in the 1990s for M2, in which we include anthropogenic O_3_ precursor emission changes along with greenhouse gas emission changes. (*A*) Changes in mean 1-hr daily maximum O_3_ concentrations (ppb). (*B*) Percent changes in O_3_-related mortality.

A different pattern of results is seen under the climate change plus O_3_ precursor emissions change assumptions represented in M2 (Table 1, Figure 3). Allowing precursor emissions to grow leads to higher O_3_ increases compared with the climate-only case outside the urban core region, but lower O_3_ concentrations in the urban core counties (Figure 3A). This reduction in urban O_3_ likely reflects the nonlinearity of the NO_x_–O_3_ relationship (Seinfeld and Pandis 1997), where in some cases increased NO_x_ in urban core areas such as New York City may react with O_3_, thereby locally lowering O_3_ concentrations. This effect is called titration.

Estimates of O_3_-related mortality here show slightly smaller impacts than were estimated under climate change alone (Table 1). The spatial pattern of mortality impacts follows a distribution similar to that of O_3_ concentrations, but with the added effects of population density (Figure 3B), because areas with relatively low population density coincide with the areas of greatest O_3_ increases, whereas areas with relatively high population density coincide with counties for which O_3_ concentrations slightly diminish. Estimated median O_3_-related summer mortality across the region increased by 4.4% above 1990s estimates.

### Sensitivity analyses.

**Figure 4:**
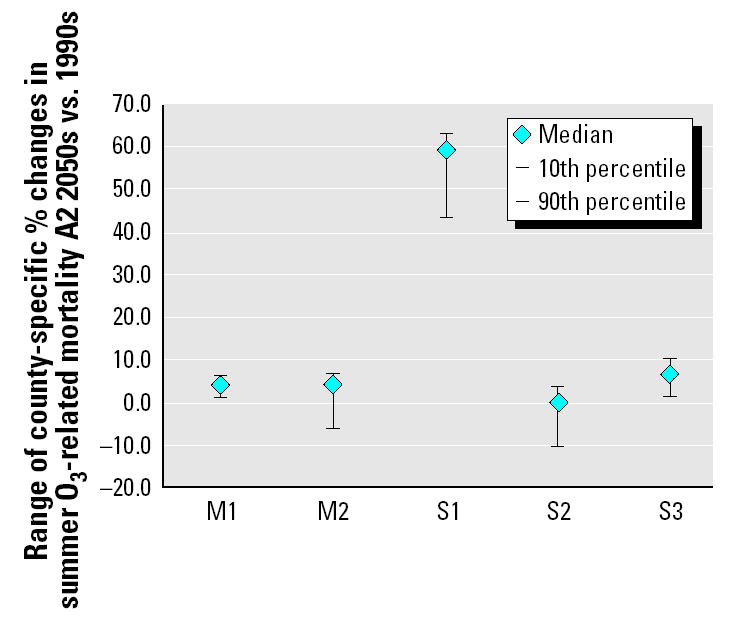
Range of projected county-specific percent increases in summer O_3_-related mortality under mortality assessments (M1, M2) and sensitivity analyses (S1–S3). M1: climate only; M2: climate and anthropogenic emissions; S1: climate, anthropogenic emissions, and population; S2: anthropogenic emissions only; S3: climate only plus minimum threshold.

The effects of varying model assumptions of individual O_3_ impacts are shown in Figure 4, which plots the median, 10th percentile, and 90th percentile of the distribution of county percent changes in mortality under each set of assumptions. The two primary mortality assessments appear as the leftmost two plots on the graph (M1–M2), followed by the series of sensitivity tests S1–S3 in the center. Sensitivity analysis S1 shows that population growth accounts for almost all of the mortality increases in the “full A2” O_3_ model simulations. In the second sensitivity analysis (S2), which considers the mortality effect of letting only anthropogenic O_3_ precursor emissions increase in the 2050s, the relative increase in mortality projections fell slightly below the M1 base case, owing to diminished O_3_ concentrations in the 2050s in the most densely populated urban core areas. The third sensitivity analysis (S3) applies an O_3_ threshold value of 20 ppb to evaluate regional mortality in both the 1990s versus 2050s and found a slightly greater percent increase in regional summer O_3_ deaths. This result was driven by a decrease in calculated mortality in the 1990s that was larger than the decrease in the 2050s under the threshold assumption.

## Discussion

Results of our analyses illustrate how integrated models can be used to assess potential impacts of climate change at regionally relevant spatial scales, suggesting that, under a variety of assumptions, climate change alone could increase regional summer O_3_-related mortality by a median 4.5% in the 2050s compared with the 1990s. These assumptions do not include the effect of projected population growth. When a more fully elaborated picture of the likely regional future was evaluated—that is, including population growth and anthropogenic O_3_ precursor emissions increases—much greater changes in summer mortality are projected: Regional summer O_3_-related mortality would increase by a median 59.9% in the 2050s compared with the 1990s. These larger impacts are dominated by the growth in population at risk.

The relatively fine spatial resolution afforded by the NYCHP model system projected spatially heterogeneous regional changes in episodic high O_3_ in coming decades. We applied O_3_ concentrations that were spatially interpolated from the 36-km model simulations to each of the 31 counties’ geographic centroids in the mortality risk assessment. This enabled us to distinguish “hot spots” in O_3_ conditions at the county level across the New York metropolitan study area. To describe their geographic distribution, we examined differences in climate-related mortality impacts projected across urban versus suburban counties within the larger New York metropolitan region. With the effects of population and precursor emission growth omitted, the greatest percent increases in summer O_3_ concentrations and related mortality are projected in the urban core and especially in a ring of suburban counties immediately surrounding the city to the southwest and east (central Long Island). When population and precursor emissions effects are also included, one can discern that areas with relatively low population density coincide with the areas of greatest O_3_ increases. Mean summer O_3_ concentrations are lower in the 2050s than in the 1990s in the most highly urbanized counties, assuming anthropogenic precursor emission growth, yet far more people are exposed here and thus mortality still increases. In future analyses from the NYCHP, we will progress to finer spatial resolutions (12 and 4 km) and include temperature-related mortality, to discern locations of vulnerable communities whose health may be most affected by climate change in the next 50 years. Beyond the immediate New York metropolitan region, the projected effects of climate changes on O_3_ concentrations and related mortality may show different patterns, owing to different overall distributions of urbanization, precursor emissions areas, and population across the eastern United States.

The sensitivity analyses showed that population growth has the largest effect on projections of changing summer O_3_-related mortality, greater than the isolated effect of climate change alone upon O_3_ concentrations and related mortality. The application of an O_3_ threshold leads to slightly greater percent increases in O_3_-related deaths than does application of the zero-threshold model, because a threshold removes the mortality effect of the days with the lowest O_3_ in both the 1990s and the 2050s. The 1990s summers had a higher proportion of below-threshold days; thus, the comparative percent increase in 2050s mortality is larger than under a no-threshold model. The effect of increasing O_3_ precursor emissions in the absence of climate change is to slightly diminish regional mortality, because summer O_3_ concentrations decrease because of the titration effect in the most densely populated urban counties. These sensitivity results illustrate the impacts of some of the uncertainties inherent in a risk assessment of this kind. Other sources of uncertainty exist that have not been included here, such as alternative modeling approaches for climate and air quality, methods for estimation of baseline summer season mortality rates, the assumption that O_3_ impacts occur only in summer, and possible modification of the O_3_–mortality relationship in the future if more households acquire air conditioning. A full uncertainty analysis was beyond the scope of the present report.

This study is the first to apply fully down-scaled global-to-regional climate model outputs to project future-year O_3_ concentrations for public health impacts assessments. The NYCHP integrates the work of health professionals with the work of air quality modelers and climate scientists and applies a linked model system to project regional mortality for a major metropolitan area of the United States. The daily simulations from the regional meteorology and air quality models at 36-km spatial resolution allowed for estimation of the public health impacts of climate change at local scales potentially useful to health care infrastructure planning. The temporal resolution of the linked model system outputs allowed us to apply CRFs from the epidemiologic literature for acute (daily) exposures and responses. The use of simulated O_3_ concentrations from the 36-km resolution atmospheric chemistry model yields more detail than that afforded by air monitoring across the 31-county New York metropolitan region. Validation of the fluctuations in surface temperature and O_3_ concentrations simulated by MM5 and CMAQ showed good agreement with 1990s observations (Hogrefe et al. 2004); hence, the integrated model system presents a useful method for studying regional climate-related changes.

Although a few previous studies have assessed O_3_ health impacts under climate change assumptions, none have used global-to-regional downscaled climate models to project O_3_ concentrations for health impacts assessment. Kleinman and Lipfert (1996) considered possible effects of climate change by simply assuming a 2°C temperature increase and evaluating its effect on O_3_ concentrations and associated mortality in the New York City area. Kalkstein and Greene (1997) were among the first to apply GCM model simulations for the 2020s and 2050s to heat-related mortality projections in 44 large U.S. cities but did not assess air quality impacts. Davis et al. (1997) considered the possible effect that climate-control policies could have on particulate air quality and associated mortality, using two possible scenarios of CO_2_ emissions but did not apply full GCM or RCM model simulations. Anderson HR et al. (2001) have projected potential O_3_-related mortality impacts associated with climate change projections for the United Kingdom in the 2020s, 2050s, and 2080s but used a GCM simulation that could not project in detail potential geographic variations in O_3_ concentrations. Two studies that used RCM simulations of climate change (Dessai 2003; McMichael et al. 2003) projected temperature-related mortality changes but not changes in mortality related to air pollution.

For scenario-based, integrated health risk assessments, there are several sources of uncertainty in estimating future impacts. The climate and air quality models used here introduce uncertainty, yet their simulations can be compared with meteorologic data to find the degree to which the models capture the observations. Furthermore, where changes are being assessed, some model biases are likely to cancel out. The O_3_ simulations we ran did not take into account, via changed boundary conditions, possible changes in air quality outside of our modeling domain. Recent work by our group suggests that these effects may be of importance in O_3_ formation equal to those related to more local changes (Hogrefe et al., in press). Mortality rates change in response to many demographic, social, behavioral, and political factors regarding individual and group health and access to health care. The climate–human health relationship within a given geography and population may change over time if populations acclimate and/or adapt to changing conditions. Part of the interdisciplinary process involved in downscaling from global to local impacts involves simplifications in each team’s modeling methods. This simplification introduces its own additional uncertainty to the results that follow. From a health science perspective, using a variety of modeling assumptions and assessing the range of results is one method for expressing uncertainty. By anticipating the range of possible impacts, the range of possibilities suggested by each scenario’s environmental, technologic, demographic, socioeconomic, and political story line can be examined. Uncertainty in the mortality risk estimates was expressed using the 95% CIs from the CRFs extracted from the epidemiologic literature.

These mortality projections do not take into account the possible effects of acclimatization or adaptive measures by the regional population. As a behavioral adaptation, the use of air conditioning could appreciably ameliorate exposures to O_3_ as well as to heat stress (O’Neill 2003; Rogot et al. 1992), because air-conditioned homes typically have lower outdoor air exchange rates than do residences without air conditioning that rely instead on open windows for ventilation (Janssen et al. 2002). In all likelihood, there will be a lag between periods of increasing environmental stress and behavioral adaptation; thus a “leading edge” of increased mortality before adaptation and/or acclimatization occurs. Furthermore, the increasingly pervasive use of air conditioners will present a potentially damaging positive feedback with climate change. Because these are highly energy-consumptive appliances, more electrical demand will occur on the hottest summer days, generating more airborne emissions from power plants and more urban waste heat from air conditioners. As evidenced during the 2003 eastern U.S. blackout, air conditioning can also sometimes be interrupted on the hottest days, owing to the increased peak demand load, and air conditioning may not really be an appropriate “fix” for adapting to climate change.

We did not consider the impacts of longer-duration O_3_ events or heat waves upon regional mortality. These will be considered in a separate, future report that compares temperature-related health effects from the GISS GCM versus MM5 RCM model outputs with the O_3_-related impacts (Knowlton K., unpublished data). The B2 (slower growth) scenario family from the IPCC *SRES* will also be evaluated as an alternative to A2, and the effects of land use changes will be included in future health impact reports.

Because of the limited scope of the project and available baseline health data, we assessed only mortality impacts in the present study. Because many other health outcomes are known to be associated with O_3_ exposures (Kinney 1999), our analysis is likely to have yielded underestimates of O_3_ impacts on health. The CRF used in the O_3_ mortality analysis (Thurston and Ito 2001) controlled for the effects of temperature upon mortality. Thus, the O_3_-related mortality estimates should not be confounded by temperature effects. The regional population’s age structure will undoubtedly change in future years, in ways that are difficult to project. The New York region has experienced appreciable immigration in recent decades that is projected to continue, along with a proportional increase in the percentage of people ≥ 65 years of age through the 2020s (U.S. Census Bureau 1996). Changes in age structure could affect the relative increase in future summer mortality in the 2020s and beyond to the 2050s, because the elderly are among those most vulnerable to heat stress and O_3_ impacts.

With the fourth assessment report of the IPCC scheduled to be released in 2007, there will be increasing emphasis on projecting the health impacts of climate change. The NYCHP linked modeling system may be a useful tool for conducting region-specific risk assessments of health impacts from future climate change and variability. These specific, local results can help bring consideration of the potential human health impacts of climate change into a public forum in those communities that may bear the burden of additional illness and mortality.

## Conclusions

The results of the integrated O_3_ health impacts assessment suggest that changes in climate alone resulting from growth in greenhouse gas emissions could cause a 4.5% increase in the number of summer O_3_-related deaths across the New York metropolitan region by the 2050s. When the additional effects of O_3_ precursor emission increases are included, a 4.4% median increase in the number of summer O_3_-related deaths across the New York metropolitan region is projected for the 2050s. O_3_ projections for the 2050s show that counties with the highest percent increases in O_3_ mortality in the 2050s, relative to the 1990s, spread beyond the urban core into less densely populated suburban counties in New Jersey, southern Connecticut, and eastern Long Island. Sensitivity analyses showed that population growth assumptions had a dominant influence over future projections of mortality related to O_3_.

## Figures and Tables

**Table 1:** Estimated county-level O_3_ concentrations and associated mortality in the 1990s and 2050s for M1 (climate only) and M2 (both climate and anthropogenic O_3_ precursor changes).

		1990s		2050s climate (M1)				2050s climate + precursors (M2)			
County	State	O_3_*^a^*	O_3_ mortality*^b^*	O_3_	ΔO_3_*^c^*	O_3_ mortality	% Δmortality*^d^*	O_3_	ΔO_3_	O_3_ mortality	% Δmortality
Fairfield	CT	61.3	57 (33–82)	63.3	2.0	59 (34–85)	3.3	64.7	3.3	60 (35–87)	5.5
Litchfield	CT	59.4	12 (7–17)	60.4	0.9	12 (7–17)	1.6	62.9	3.5	12 (7–18)	5.9
New Haven	CT	62.1	61 (35–88)	64.5	2.5	63 (36–91)	4.0	65.8	3.7	64 (37–93)	6.1
Bergen	NJ	49.7	50 (29–72)	51.9	2.2	52 (30–75)	4.5	49.0	−0.7	49 (28–71)	−1.5
Essex	NJ	52.0	52 (30–75)	54.3	2.3	54 (31–78)	4.5	51.9	−0.1	52 (30–75)	−0.2
Hudson	NJ	44.1	31 (18–45)	46.2	2.2	33 (19–47)	5.0	41.3	−2.8	29 (17–42)	−6.3
Hunterdon	NJ	64.3	6 (3–9)	67.2	2.9	6 (4–9)	4.6	68.6	4.3	6 (4–9)	6.8
Mercer	NJ	62.6	25 (14–36)	66.9	4.3	26 (15–38)	7.0	66.6	4.0	26 (15–38)	6.5
Middlesex	NJ	55.4	41 (23–58)	58.9	3.5	43 (25–62)	6.4	56.8	1.4	42 (24–60)	2.6
Monmouth	NJ	54.8	38 (22–54)	58.1	3.3	40 (23–57)	6.2	56.5	1.7	39 (22–56)	3.2
Morris	NJ	61.9	26 (15–37)	64.1	2.2	27 (15–39)	3.7	64.9	3.1	27 (16–39)	5.0
Ocean	NJ	62.6	55 (31–79)	65.9	3.3	57 (33–83)	5.4	68.6	6.0	60 (34–86)	9.7
Passaic	NJ	59.7	33 (19–47)	61.2	1.5	33 (19–48)	2.5	62.0	2.3	34 (19–49)	3.9
Somerset	NJ	64.5	17 (10–24)	67.9	3.4	18 (10–25)	5.4	68.7	4.2	18 (10–26)	6.6
Sussex	NJ	60.9	7 (4–11)	61.6	0.7	8 (4–11)	1.2	63.7	2.8	8 (4–11)	4.6
Union	NJ	52.1	33 (19–47)	54.8	2.7	35 (20–50)	5.3	52.1	0.0	33 (19–47)	0.0
Warren	NJ	63.2	7 (4–10)	65.0	1.8	7 (4–11)	2.9	66.8	3.6	8 (4–11)	5.9
Bronx	NY	49.7	81 (46–116)	52.1	2.4	85 (49–122)	4.9	48.8	−0.9	79 (45–114)	−1.9
Dutchess	NY	59.8	17 (10–25)	60.3	0.5	17 (10–25)	0.9	62.8	3.0	18 (10–26)	5.1
Kings	NY	44.1	123 (71–176)	46.5	2.4	129 (74–186)	5.6	41.2	−2.9	115 (66–164)	−6.6
Nassau	NY	56.6	83 (48–119)	60.1	3.4	88 (51–127)	6.2	57.7	1.1	85 (49–122)	1.9
New York	NY	44.7	78 (45–113)	46.8	2.1	82 (47–118)	4.8	42.0	−2.7	74 (42–106)	−6.1
Orange	NY	60.0	20 (11–28)	60.3	0.3	20 (11–28)	0.4	62.6	2.6	20 (12–29)	4.3
Putnam	NY	61.0	5 (3–7)	61.9	0.9	5 (3–7)	1.6	63.9	2.9	5 (3–7)	4.8
Queens	NY	47.9	120 (69–172)	50.4	2.5	126 (73–181)	5.3	46.1	−1.8	115 (66–166)	−3.7
Richmond	NY	43.0	21 (12–30)	45.6	2.6	22 (13–32)	6.1	39.9	−3.1	19 (11–28)	−7.2
Rockland	NY	59.6	16 (9–23)	61.5	1.9	17 (9–24)	3.2	62.1	2.6	17 (10–24)	4.4
Suffolk	NY	59.0	84 (48–121)	61.5	2.5	87 (50–126)	4.3	61.5	2.5	87 (50–126)	4.3
Sullivan	NY	58.0	6 (3–8)	58.3	0.4	6 (3–9)	0.6	60.9	3.0	6 (4–9)	5.2
Ulster	NY	57.6	12 (7–18)	58.1	0.5	12 (7–18)	0.8	60.7	3.1	13 (7–19)	5.4
Westchester	NY	59.9	61 (35–88)	62.5	2.6	64 (37–92)	4.3	62.9	3.0	64 (37–93)	5.0

a Mean summer 1-hr daily maximum O_3_ concentration in ppb.

b Mean summer O_3_-related mortality typical of decade (95% CI).

c Change in mean summer 1-hr daily maximum O_3_ concentration, 2050s versus 1990s.

d Percent change in typical summer O_3_-related mortality, 2050s versus 1990s.
